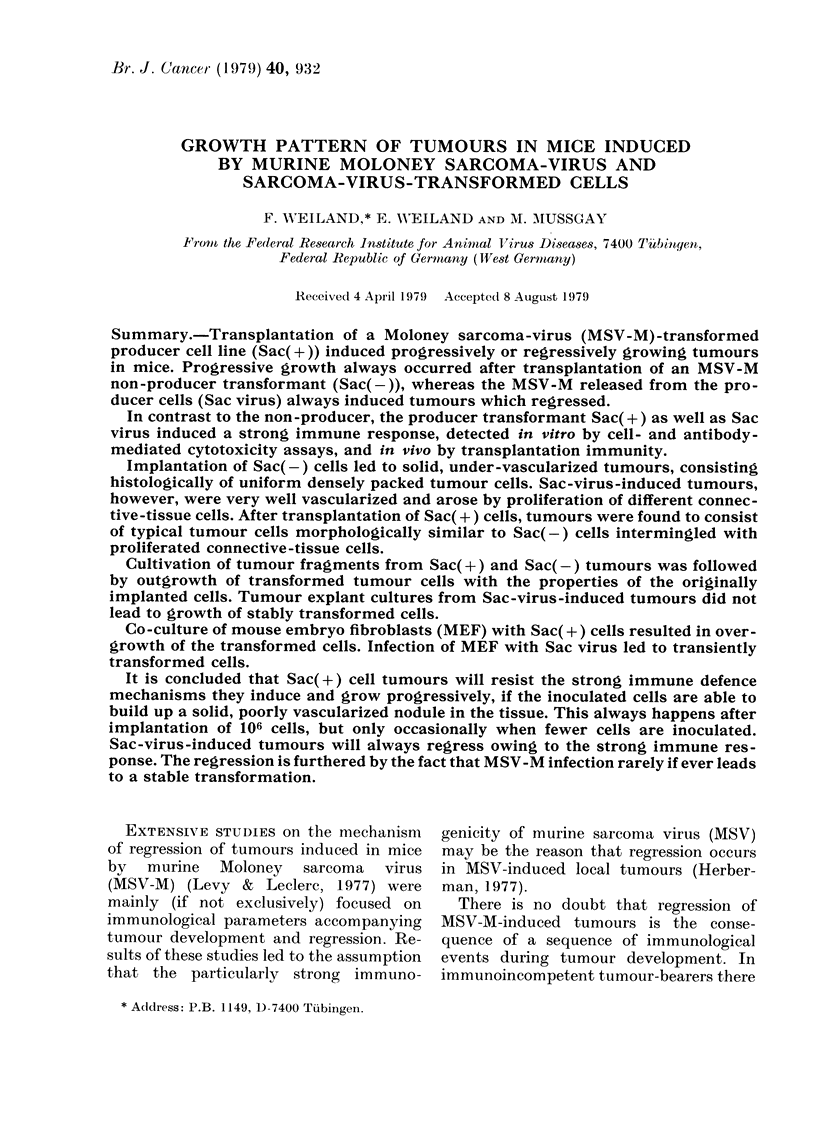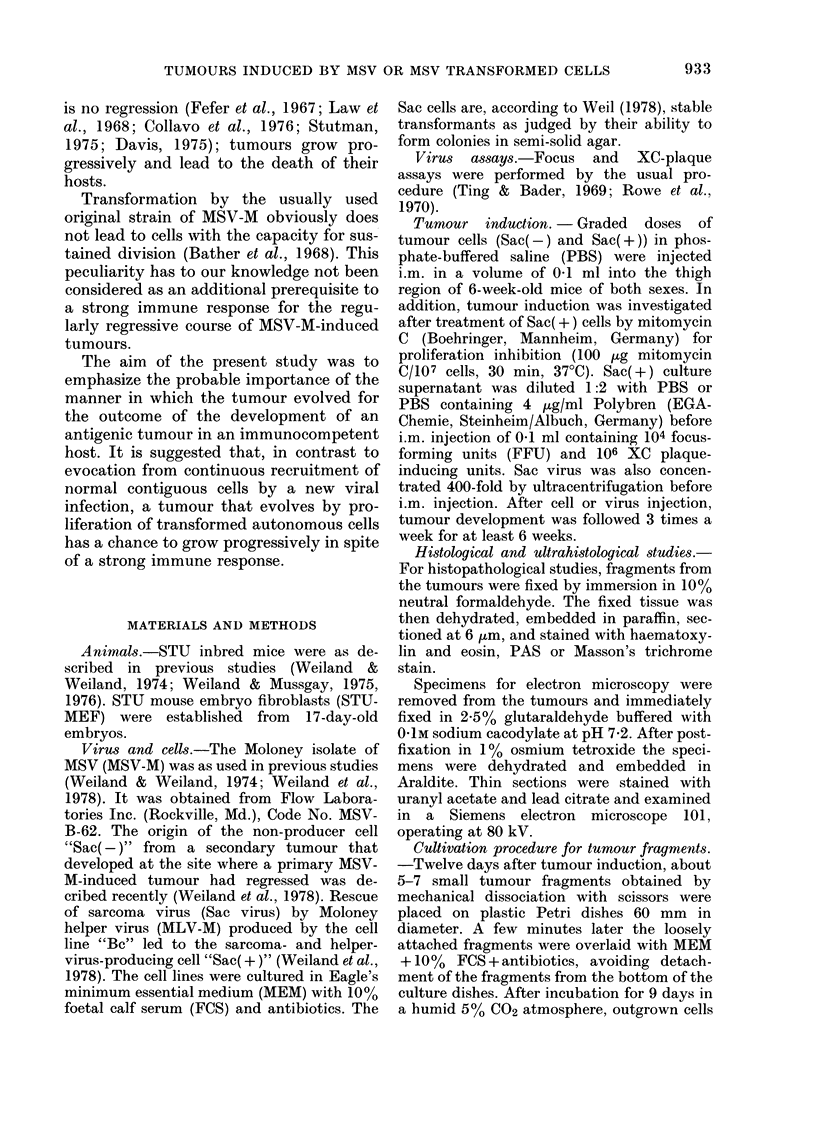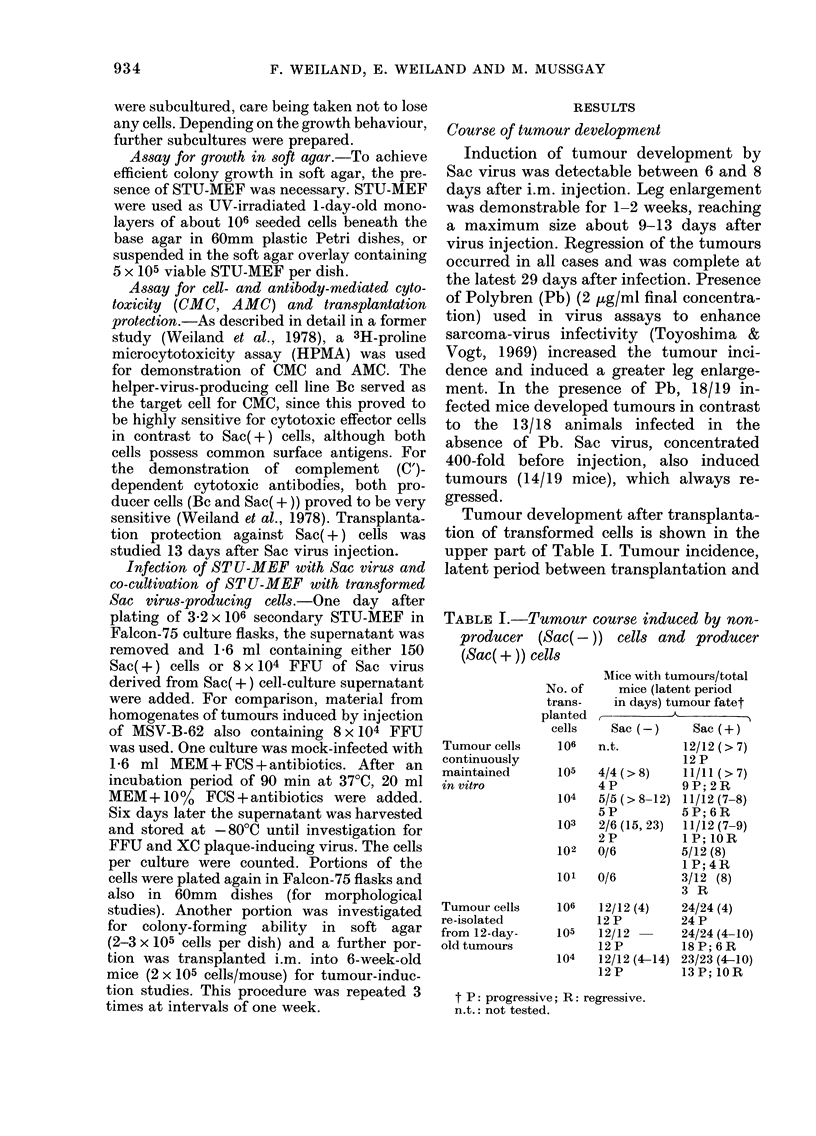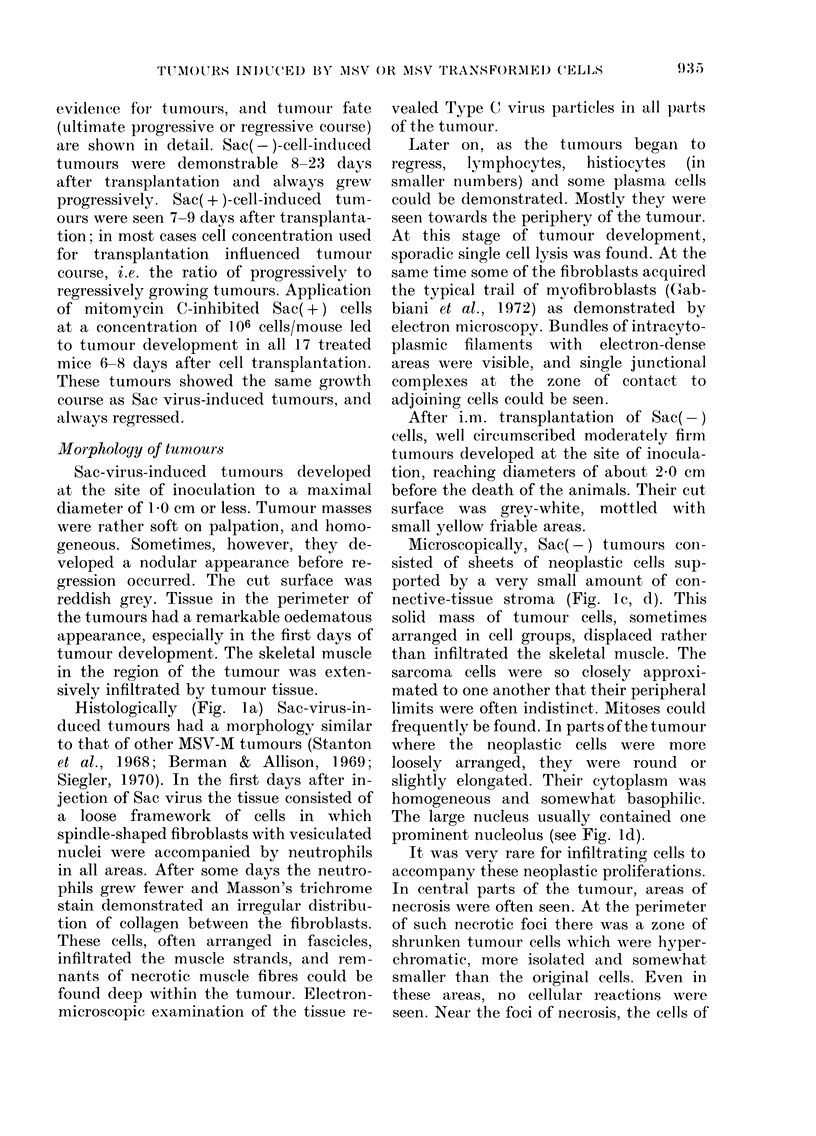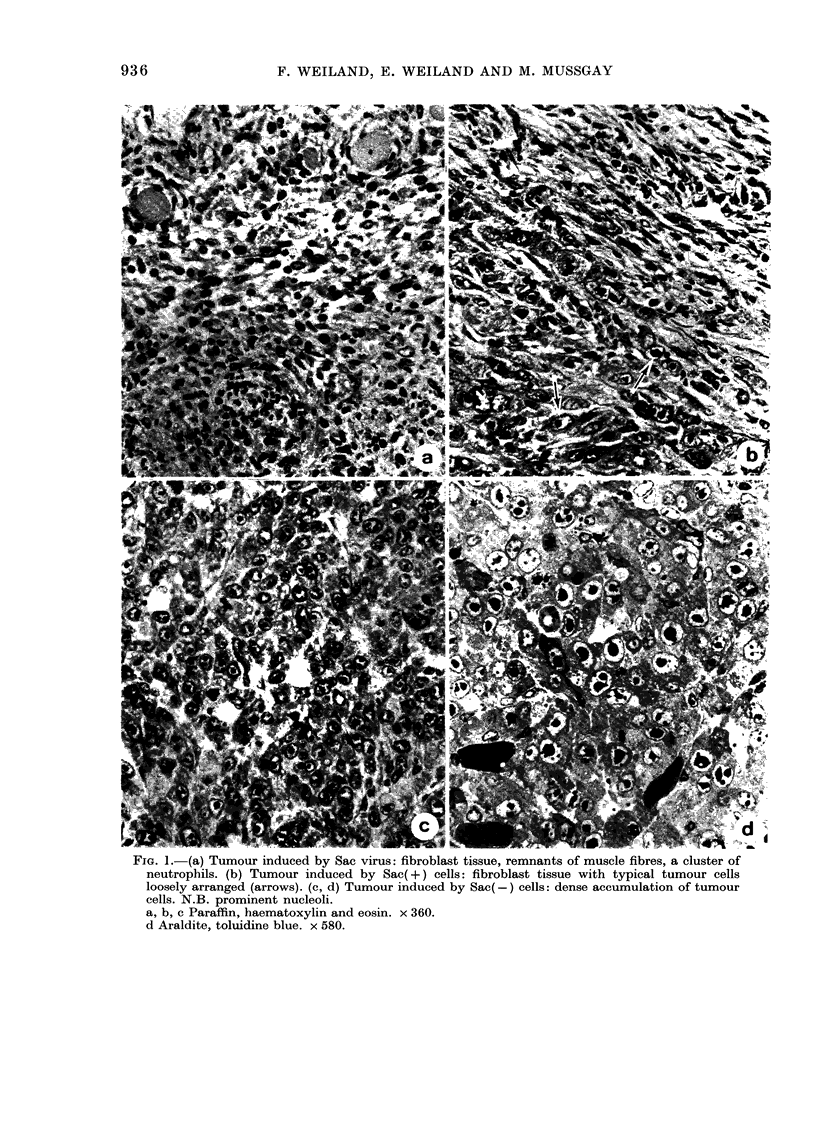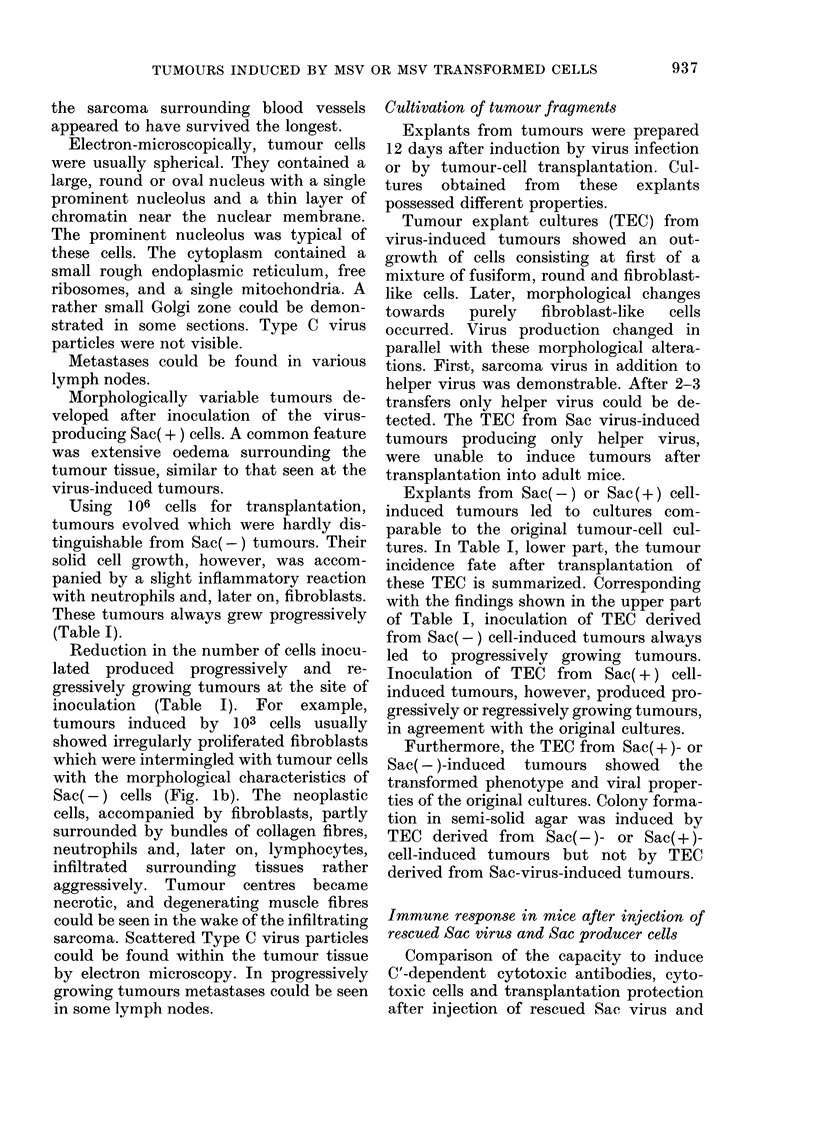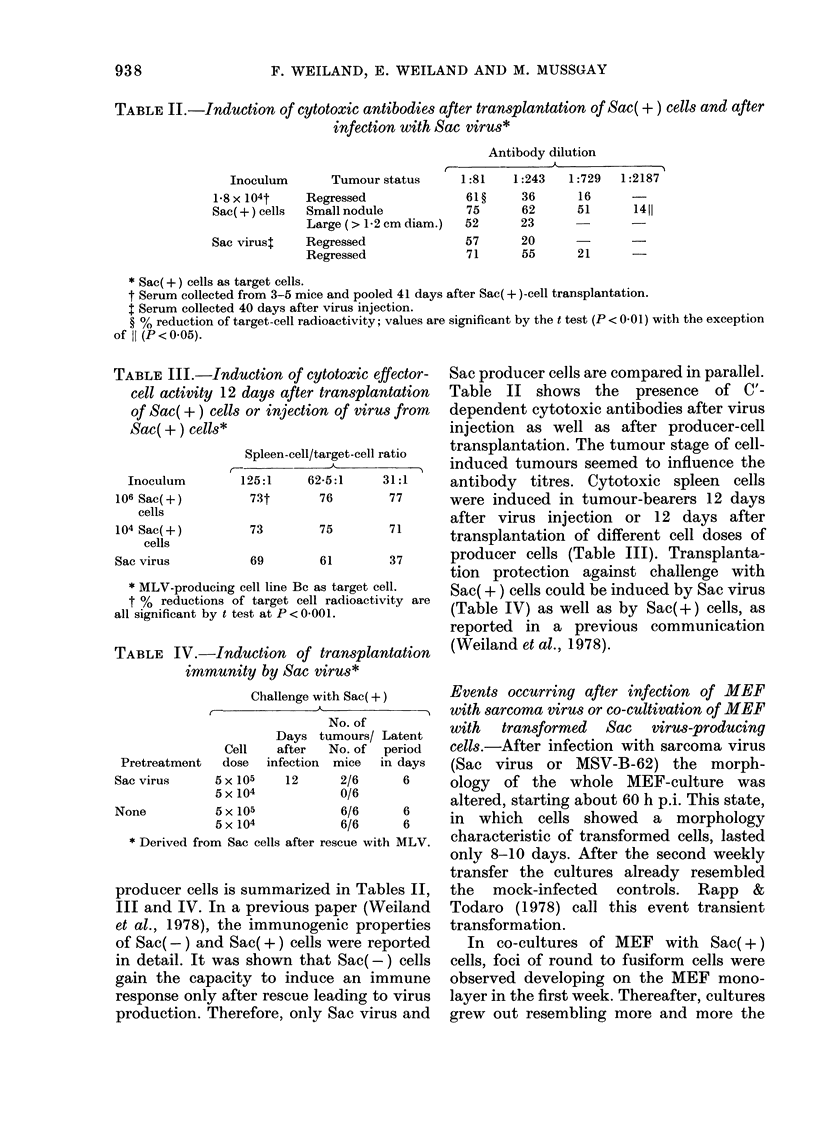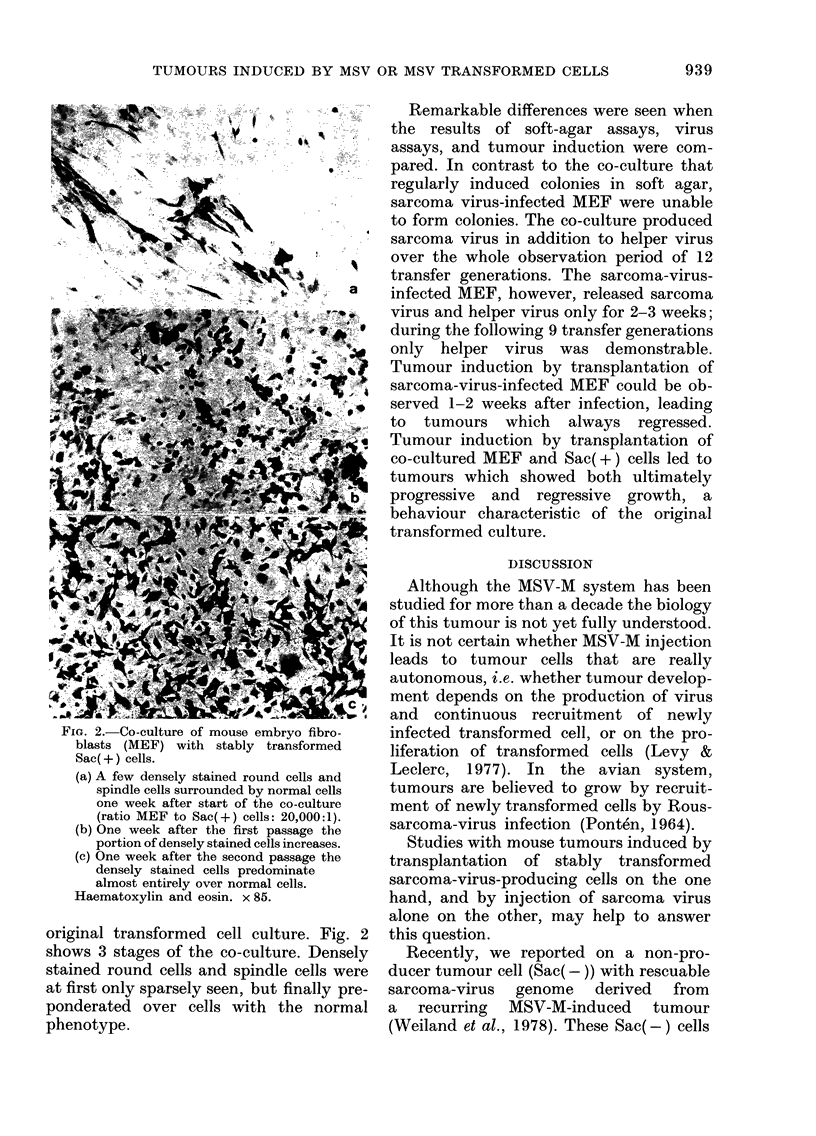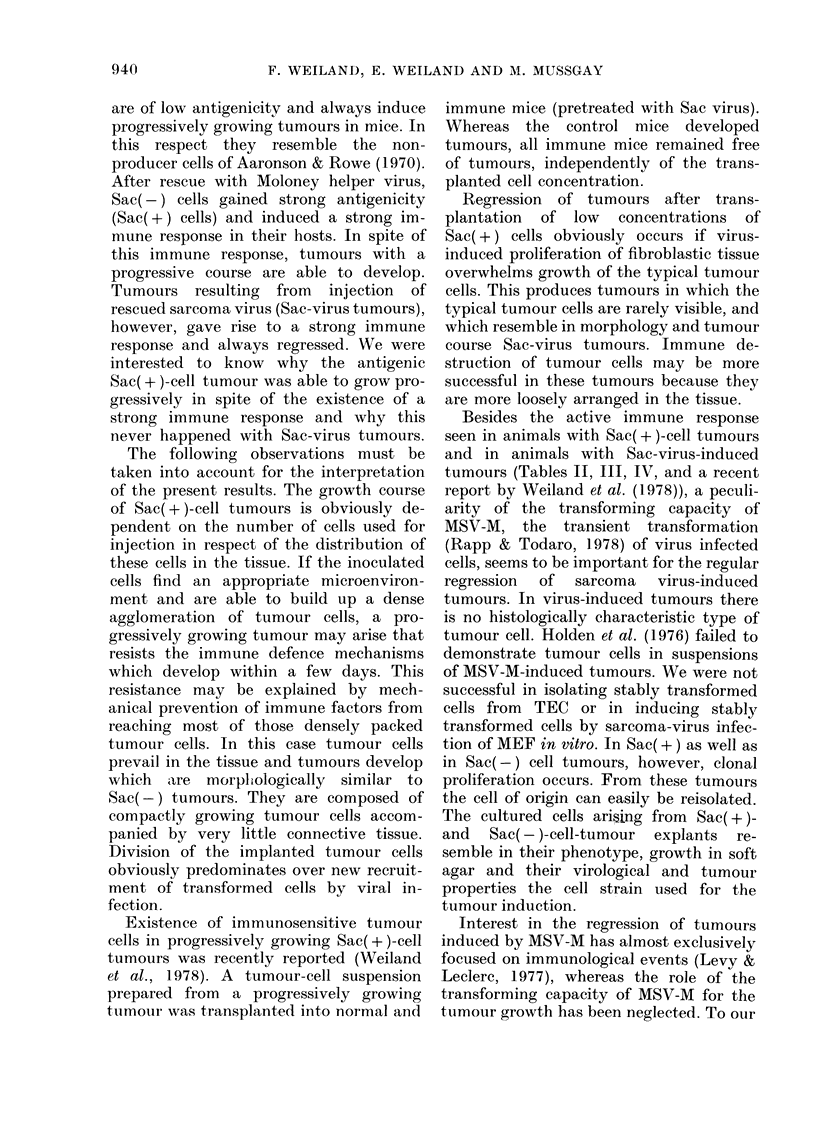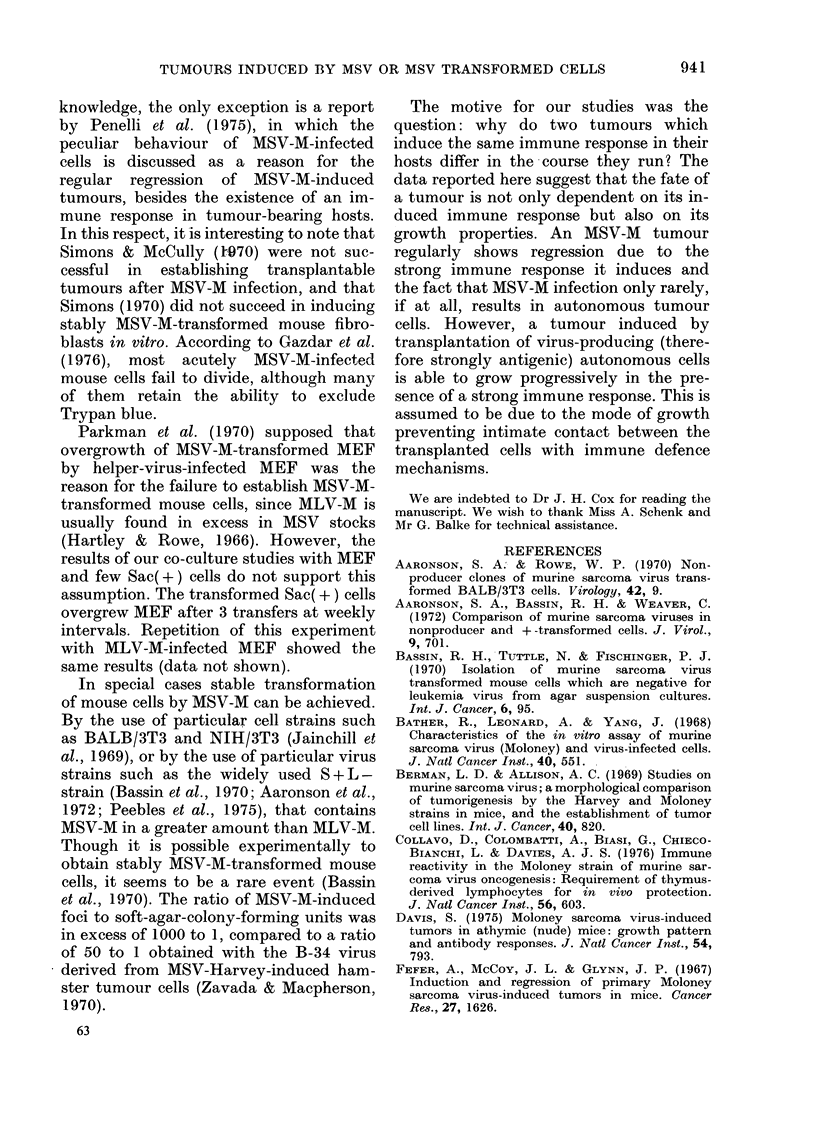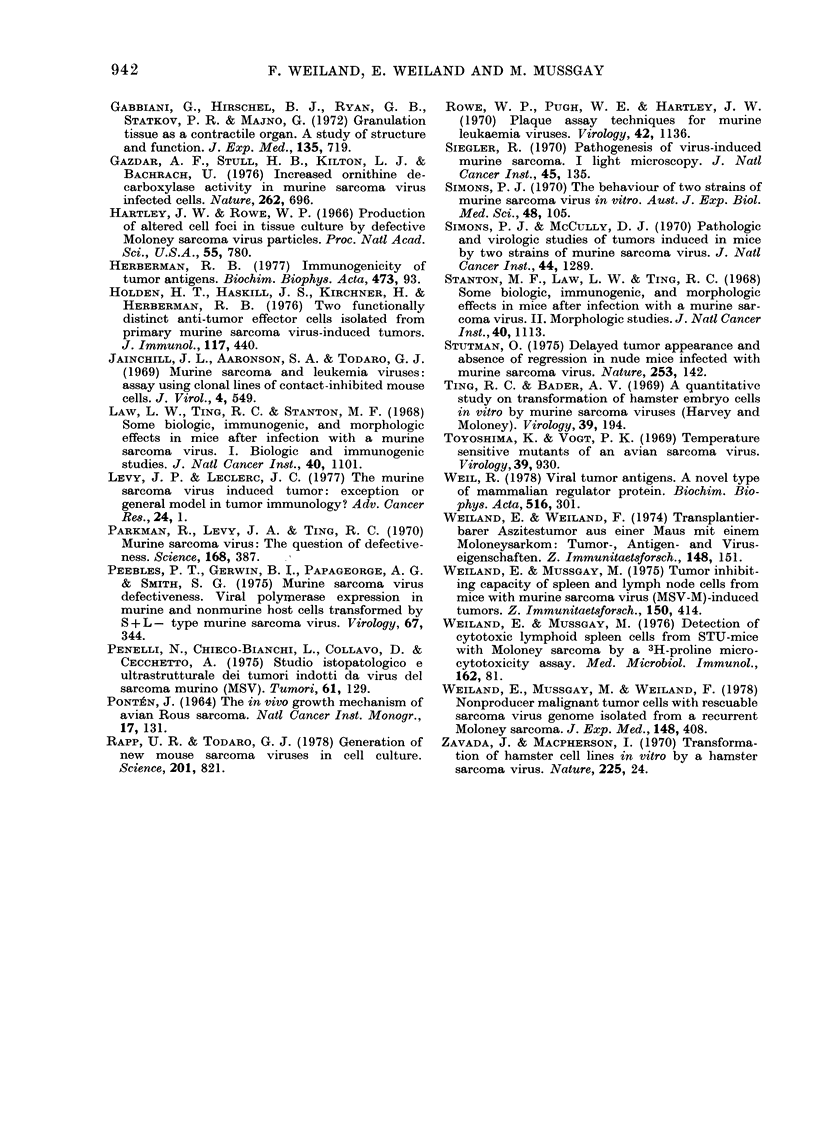# Growth pattern of tumours in mice induced by murine Moloney sarcoma-virus and sarcoma-virus-transformed cells.

**DOI:** 10.1038/bjc.1979.288

**Published:** 1979-12

**Authors:** F. Weiland, E. Weiland, M. Mussgay

## Abstract

**Images:**


*Br. J. Cancer* (1979) **40**, 932

### GROWTH PATTERN OF TUMOURS IN MICE INDUCED BY MURINE MOLONEY SARCOMA-VIRUS AND SARCOMA-VIRUS-TRANSFORMED CELLS

F. WEILAND,* E. WEILAND AND M. MUSSGAY

*From the Federal Research Institute for Animal Virus Diseases, 7400 Tübingen, Federal Republic of Germany (West Germany)*

Received 4 April 1979 Accepted 8 August 1979

**Summary.—Transplantation of a Moloney sarcoma-virus (MSV-M)-transformed producer cell line (Sac(+)) induced progressively or regressively growing tumours in mice. Progressive growth always occurred after transplantation of an MSV-M non-producer transformant (Sac(−)), whereas the MSV-M released from the producer cells (Sac virus) always induced tumours which regressed.**

**In contrast to the non-producer, the producer transformant Sac(+) as well as Sac virus induced a strong immune response, detected *in vitro* by cell- and antibody-mediated cytotoxicity assays, and *in vivo* by transplantation immunity.**

**Implantation of Sac(−) cells led to solid, under-vascularized tumours, consisting histologically of uniform densely packed tumour cells. Sac-virus-induced tumours, however, were very well vascularized and arose by proliferation of different connective-tissue cells. After transplantation of Sac(+) cells, tumours were found to consist of typical tumour cells morphologically similar to Sac(−) cells intermingled with proliferated connective-tissue cells.**

**Cultivation of tumour fragments from Sac(+) and Sac(−) tumours was followed by outgrowth of transformed tumour cells with the properties of the originally implanted cells. Tumour explant cultures from Sac-virus-induced tumours did not lead to growth of stably transformed cells.**

**Co-culture of mouse embryo fibroblasts (MEF) with Sac(+) cells resulted in overgrowth of the transformed cells. Infection of MEF with Sac virus led to transiently transformed cells.**

**It is concluded that Sac(+) cell tumours will resist the strong immune defence mechanisms they induce and grow progressively, if the inoculated cells are able to build up a solid, poorly vascularized nodule in the tissue. This always happens after implantation of $10^6$ cells, but only occasionally when fewer cells are inoculated. Sac-virus-induced tumours will always regress owing to the strong immune response. The regression is furthered by the fact that MSV-M infection rarely if ever leads to a stable transformation.**

EXTENSIVE STUDIES on the mechanism of regression of tumours induced in mice by murine Moloney sarcoma virus (MSV-M) (Levy & Leclerc, 1977) were mainly (if not exclusively) focused on immunological parameters accompanying tumour development and regression. Results of these studies led to the assumption that the particularly strong immunogenicity of murine sarcoma virus (MSV) may be the reason that regression occurs in MSV-induced local tumours (Herberman, 1977).

There is no doubt that regression of MSV-M-induced tumours is the consequence of a sequence of immunological events during tumour development. In immunoincompetent tumour-bearers there

* Address: P.B. 1149, D-7400 Tübingen.

is no regression (Fefer *et al.*, 1967; Law *et al.*, 1968; Collavo *et al.*, 1976; Stutman, 1975; Davis, 1975); tumours grow progressively and lead to the death of their hosts.

Transformation by the usually used original strain of MSV-M obviously does not lead to cells with the capacity for sustained division (Bather *et al.*, 1968). This peculiarity has to our knowledge not been considered as an additional prerequisite to a strong immune response for the regularly regressive course of MSV-M-induced tumours.

The aim of the present study was to emphasize the probable importance of the manner in which the tumour evolved for the outcome of the development of an antigenic tumour in an immunocompetent host. It is suggested that, in contrast to evocation from continuous recruitment of normal contiguous cells by a new viral infection, a tumour that evolves by proliferation of transformed autonomous cells has a chance to grow progressively in spite of a strong immune response.

### MATERIALS AND METHODS

*Animals*.—STU inbred mice were as described in previous studies (Weiland & Weiland, 1974; Weiland & Mussgay, 1975, 1976). STU mouse embryo fibroblasts (STU-MEF) were established from 17-day-old embryos.

*Virus and cells*.—The Moloney isolate of MSV (MSV-M) was as used in previous studies (Weiland & Weiland, 1974; Weiland *et al.*, 1978). It was obtained from Flow Laboratories Inc. (Rockville, Md.), Code No. MSV-B-62. The origin of the non-producer cell "Sac(−)" from a secondary tumour that developed at the site where a primary MSV-M-induced tumour had regressed was decribed recently (Weiland *et al.*, 1978). Rescue of sarcoma virus (Sac virus) by Moloney helper virus (MLV-M) produced by the cell line "Bc" led to the sarcoma- and helper-virus-producing cell "Sac(+)" (Weiland *et al.*, 1978). The cell lines were cultured in Eagle's minimum essential medium (MEM) with 10% foetal calf serum (FCS) and antibiotics. The Sac cells are, according to Weil (1978), stable transformants as judged by their ability to form colonies in semi-solid agar.

*Virus assays*.—Focus and XC-plaque assays were performed by the usual procedure (Ting & Bader, 1969; Rowe *et al.*, 1970).

*Tumour induction*. — Graded doses of tumour cells (Sac(−) and Sac(+)) in phosphate-buffered saline (PBS) were injected i.m. in a volume of 0·1 ml into the thigh region of 6-week-old mice of both sexes. In addition, tumour induction was investigated after treatment of Sac(+) cells by mitomycin C (Boehringer, Mannheim, Germany) for proliferation inhibition (100 $\mu$g mitomycin C/$10^7$ cells, 30 min, 37°C). Sac(+) culture supernatant was diluted 1:2 with PBS or PBS containing 4 $\mu$g/ml Polybren (EGA-Chemie, Steinheim/Albuch, Germany) before i.m. injection of 0·1 ml containing $10^4$ focus-forming units (FFU) and $10^6$ XC plaque-inducing units. Sac virus was also concentrated 400-fold by ultracentrifugation before i.m. injection. After cell or virus injection, tumour development was followed 3 times a week for at least 6 weeks.

*Histological and ultrahistological studies*.—For histopathological studies, fragments from the tumours were fixed by immersion in 10% neutral formaldehyde. The fixed tissue was then dehydrated, embedded in paraffin, sectioned at 6 $\mu$m, and stained with haematoxylin and eosin, PAS or Masson's trichrome stain.

Specimens for electron microscopy were removed from the tumours and immediately fixed in 2·5% glutaraldehyde buffered with 0·1M sodium cacodylate at pH 7·2. After post-fixation in 1% osmium tetroxide the specimens were dehydrated and embedded in Araldite. Thin sections were stained with uranyl acetate and lead citrate and examined in a Siemens electron microscope 101, operating at 80 kV.

*Cultivation procedure for tumour fragments*.—Twelve days after tumour induction, about 5–7 small tumour fragments obtained by mechanical dissociation with scissors were placed on plastic Petri dishes 60 mm in diameter. A few minutes later the loosely attached fragments were overlaid with MEM +10% FCS+antibiotics, avoiding detachment of the fragments from the bottom of the culture dishes. After incubation for 9 days in a humid 5% $CO_2$ atmosphere, outgrown cells were subcultured, care being taken not to lose any cells. Depending on the growth behaviour, further subcultures were prepared.

*Assay for growth in soft agar.*—To achieve efficient colony growth in soft agar, the presence of STU-MEF was necessary. STU-MEF were used as UV-irradiated 1-day-old monolayers of about $10^6$ seeded cells beneath the base agar in 60mm plastic Petri dishes, or suspended in the soft agar overlay containing $5 \times 10^5$ viable STU-MEF per dish.

*Assay for cell- and antibody-mediated cytotoxicity (CMC, AMC) and transplantation protection.*—As described in detail in a former study (Weiland *et al.*, 1978), a $^3$H-proline microcytotoxicity assay (HPMA) was used for demonstration of CMC and AMC. The helper-virus-producing cell line Bc served as the target cell for CMC, since this proved to be highly sensitive for cytotoxic effector cells in contrast to Sac(+) cells, although both cells possess common surface antigens. For the demonstration of complement (C′)-dependent cytotoxic antibodies, both producer cells (Bc and Sac(+)) proved to be very sensitive (Weiland *et al.*, 1978). Transplantation protection against Sac(+) cells was studied 13 days after Sac virus injection.

*Infection of STU-MEF with Sac virus and co-cultivation of STU-MEF with transformed Sac virus-producing cells.*—One day after plating of $3{\cdot}2 \times 10^6$ secondary STU-MEF in Falcon-75 culture flasks, the supernatant was removed and 1·6 ml containing either 150 Sac(+) cells or $8 \times 10^4$ FFU of Sac virus derived from Sac(+) cell-culture supernatant were added. For comparison, material from homogenates of tumours induced by injection of MSV-B-62 also containing $8 \times 10^4$ FFU was used. One culture was mock-infected with 1·6 ml MEM+FCS+antibiotics. After an incubation period of 90 min at 37°C, 20 ml MEM+10% FCS+antibiotics were added. Six days later the supernatant was harvested and stored at −80°C until investigation for FFU and XC plaque-inducing virus. The cells per culture were counted. Portions of the cells were plated again in Falcon-75 flasks and also in 60mm dishes (for morphological studies). Another portion was investigated for colony-forming ability in soft agar ($2$–$3 \times 10^5$ cells per dish) and a further portion was transplanted i.m. into 6-week-old mice ($2 \times 10^5$ cells/mouse) for tumour-induction studies. This procedure was repeated 3 times at intervals of one week.

### RESULTS

### *Course of tumour development*

Induction of tumour development by Sac virus was detectable between 6 and 8 days after i.m. injection. Leg enlargement was demonstrable for 1–2 weeks, reaching a maximum size about 9–13 days after virus injection. Regression of the tumours occurred in all cases and was complete at the latest 29 days after infection. Presence of Polybren (Pb) (2 μg/ml final concentration) used in virus assays to enhance sarcoma-virus infectivity (Toyoshima & Vogt, 1969) increased the tumour incidence and induced a greater leg enlargement. In the presence of Pb, 18/19 infected mice developed tumours in contrast to the 13/18 animals infected in the absence of Pb. Sac virus, concentrated 400-fold before injection, also induced tumours (14/19 mice), which always regressed.

Tumour development after transplantation of transformed cells is shown in the upper part of Table I. Tumour incidence, latent period between transplantation and

TABLE I.—*Tumour course induced by non-producer (Sac(−)) cells and producer (Sac(+)) cells*

| | No. of transplanted cells | Mice with tumours/total mice (latent period in days) tumour fate† | |
|---|---|---|---|
| | | Sac (−) | Sac (+) |
| Tumour cells continuously maintained *in vitro* | $10^6$ | n.t. | 12/12 (>7) 12 P |
| | $10^5$ | 4/4 (>8) 4 P | 11/11 (>7) 9 P; 2 R |
| | $10^4$ | 5/5 (>8–12) 5 P | 11/12 (7–8) 5 P; 6 R |
| | $10^3$ | 2/6 (15, 23) 2 P | 11/12 (7–9) 1 P; 10 R |
| | $10^2$ | 0/6 | 5/12 (8) 1 P; 4 R |
| | $10^1$ | 0/6 | 3/12 (8) 3 R |
| Tumour cells re-isolated from 12-day-old tumours | $10^6$ | 12/12 (4) 12 P | 24/24 (4) 24 P |
| | $10^5$ | 12/12 — 12 P | 24/24 (4–10) 18 P; 6 R |
| | $10^4$ | 12/12 (4–14) 12 P | 23/23 (4–10) 13 P; 10 R |

† P: progressive; R: regressive.
n.t.: not tested.

evidence for tumours, and tumour fate (ultimate progressive or regressive course) are shown in detail. Sac(–)-cell-induced tumours were demonstrable 8–23 days after transplantation and always grew progressively. Sac(+)-cell-induced tumours were seen 7–9 days after transplantation; in most cases cell concentration used for transplantation influenced tumour course, *i.e.* the ratio of progressively to regressively growing tumours. Application of mitomycin C-inhibited Sac(+) cells at a concentration of $10^6$ cells/mouse led to tumour development in all 17 treated mice 6–8 days after cell transplantation. These tumours showed the same growth course as Sac virus-induced tumours, and always regressed.

*Morphology of tumours*

Sac-virus-induced tumours developed at the site of inoculation to a maximal diameter of 1·0 cm or less. Tumour masses were rather soft on palpation, and homogeneous. Sometimes, however, they developed a nodular appearance before regression occurred. The cut surface was reddish grey. Tissue in the perimeter of the tumours had a remarkable oedematous appearance, especially in the first days of tumour development. The skeletal muscle in the region of the tumour was extensively infiltrated by tumour tissue.

Histologically (Fig. 1a) Sac-virus-induced tumours had a morphology similar to that of other MSV-M tumours (Stanton *et al.*, 1968; Berman & Allison, 1969; Siegler, 1970). In the first days after injection of Sac virus the tissue consisted of a loose framework of cells in which spindle-shaped fibroblasts with vesiculated nuclei were accompanied by neutrophils in all areas. After some days the neutrophils grew fewer and Masson's trichrome stain demonstrated an irregular distribution of collagen between the fibroblasts. These cells, often arranged in fascicles, infiltrated the muscle strands, and remnants of necrotic muscle fibres could be found deep within the tumour. Electron-microscopic examination of the tissue revealed Type C virus particles in all parts of the tumour.

Later on, as the tumours began to regress, lymphocytes, histiocytes (in smaller numbers) and some plasma cells could be demonstrated. Mostly they were seen towards the periphery of the tumour. At this stage of tumour development, sporadic single cell lysis was found. At the same time some of the fibroblasts acquired the typical trail of myofibroblasts (Gabbiani *et al.*, 1972) as demonstrated by electron microscopy. Bundles of intracytoplasmic filaments with electron-dense areas were visible, and single junctional complexes at the zone of contact to adjoining cells could be seen.

After i.m. transplantation of Sac(–) cells, well circumscribed moderately firm tumours developed at the site of inoculation, reaching diameters of about 2·0 cm before the death of the animals. Their cut surface was grey-white, mottled with small yellow friable areas.

Microscopically, Sac(–) tumours consisted of sheets of neoplastic cells supported by a very small amount of connective-tissue stroma (Fig. 1c, d). This solid mass of tumour cells, sometimes arranged in cell groups, displaced rather than infiltrated the skeletal muscle. The sarcoma cells were so closely approximated to one another that their peripheral limits were often indistinct. Mitoses could frequently be found. In parts of the tumour where the neoplastic cells were more loosely arranged, they were round or slightly elongated. Their cytoplasm was homogeneous and somewhat basophilic. The large nucleus usually contained one prominent nucleolus (see Fig. 1d).

It was very rare for infiltrating cells to accompany these neoplastic proliferations. In central parts of the tumour, areas of necrosis were often seen. At the perimeter of such necrotic foci there was a zone of shrunken tumour cells which were hyperchromatic, more isolated and somewhat smaller than the original cells. Even in these areas, no cellular reactions were seen. Near the foci of necrosis, the cells of

FIG. 1.—(a) Tumour induced by Sac virus: fibroblast tissue, remnants of muscle fibres, a cluster of neutrophils. (b) Tumour induced by Sac(+) cells: fibroblast tissue with typical tumour cells loosely arranged (arrows). (c, d) Tumour induced by Sac(−) cells: dense accumulation of tumour cells. N.B. prominent nucleoli.
a, b, c Paraffin, haematoxylin and eosin. × 360.
d Araldite, toluidine blue. × 580.

the sarcoma surrounding blood vessels appeared to have survived the longest.

Electron-microscopically, tumour cells were usually spherical. They contained a large, round or oval nucleus with a single prominent nucleolus and a thin layer of chromatin near the nuclear membrane. The prominent nucleolus was typical of these cells. The cytoplasm contained a small rough endoplasmic reticulum, free ribosomes, and a single mitochondria. A rather small Golgi zone could be demonstrated in some sections. Type C virus particles were not visible.

Metastases could be found in various lymph nodes.

Morphologically variable tumours developed after inoculation of the virus-producing Sac(+) cells. A common feature was extensive oedema surrounding the tumour tissue, similar to that seen at the virus-induced tumours.

Using $10^6$ cells for transplantation, tumours evolved which were hardly distinguishable from Sac(−) tumours. Their solid cell growth, however, was accompanied by a slight inflammatory reaction with neutrophils and, later on, fibroblasts. These tumours always grew progressively (Table I).

Reduction in the number of cells inoculated produced progressively and regressively growing tumours at the site of inoculation (Table I). For example, tumours induced by $10^3$ cells usually showed irregularly proliferated fibroblasts which were intermingled with tumour cells with the morphological characteristics of Sac(−) cells (Fig. 1b). The neoplastic cells, accompanied by fibroblasts, partly surrounded by bundles of collagen fibres, neutrophils and, later on, lymphocytes, infiltrated surrounding tissues rather aggressively. Tumour centres became necrotic, and degenerating muscle fibres could be seen in the wake of the infiltrating sarcoma. Scattered Type C virus particles could be found within the tumour tissue by electron microscopy. In progressively growing tumours metastases could be seen in some lymph nodes.

*Cultivation of tumour fragments*

Explants from tumours were prepared 12 days after induction by virus infection or by tumour-cell transplantation. Cultures obtained from these explants possessed different properties.

Tumour explant cultures (TEC) from virus-induced tumours showed an outgrowth of cells consisting at first of a mixture of fusiform, round and fibroblast-like cells. Later, morphological changes towards purely fibroblast-like cells occurred. Virus production changed in parallel with these morphological alterations. First, sarcoma virus in addition to helper virus was demonstrable. After 2–3 transfers only helper virus could be detected. The TEC from Sac virus-induced tumours producing only helper virus, were unable to induce tumours after transplantation into adult mice.

Explants from Sac(−) or Sac(+) cell-induced tumours led to cultures comparable to the original tumour-cell cultures. In Table I, lower part, the tumour incidence fate after transplantation of these TEC is summarized. Corresponding with the findings shown in the upper part of Table I, inoculation of TEC derived from Sac(−) cell-induced tumours always led to progressively growing tumours. Inoculation of TEC from Sac(+) cell-induced tumours, however, produced progressively or regressively growing tumours, in agreement with the original cultures.

Furthermore, the TEC from Sac(+)- or Sac(−)-induced tumours showed the transformed phenotype and viral properties of the original cultures. Colony formation in semi-solid agar was induced by TEC derived from Sac(−)- or Sac(+)-cell-induced tumours but not by TEC derived from Sac-virus-induced tumours.

*Immune response in mice after injection of rescued Sac virus and Sac producer cells*

Comparison of the capacity to induce C′-dependent cytotoxic antibodies, cytotoxic cells and transplantation protection after injection of rescued Sac virus and

TABLE II.—*Induction of cytotoxic antibodies after transplantation of Sac( + ) cells and after infection with Sac virus**

| Inoculum | Tumour status | Antibody dilution 1:81 | 1:243 | 1:729 | 1:2187 |
|---|---|---|---|---|---|
| $1·8 \times 10^4$† | Regressed | 61§ | 36 | 16 | — |
| Sac( + ) cells | Small nodule | 75 | 62 | 51 | 14‖ |
| | Large ( > 1·2 cm diam.) | 52 | 23 | — | — |
| Sac virus‡ | Regressed | 57 | 20 | — | — |
| | Regressed | 71 | 55 | 21 | — |

* Sac( + ) cells as target cells.
† Serum collected from 3–5 mice and pooled 41 days after Sac( + )-cell transplantation.
‡ Serum collected 40 days after virus injection.
§ % reduction of target-cell radioactivity; values are significant by the *t* test ($P < 0·01$) with the exception of ‖ ($P < 0·05$).

TABLE III.—*Induction of cytotoxic effector-cell activity* 12 *days after transplantation of Sac( + ) cells or injection of virus from Sac( + ) cells**

| Inoculum | Spleen-cell/target-cell ratio 125:1 | 62·5:1 | 31:1 |
|---|---|---|---|
| $10^6$ Sac( + ) cells | 73† | 76 | 77 |
| $10^4$ Sac( + ) cells | 73 | 75 | 71 |
| Sac virus | 69 | 61 | 37 |

* MLV-producing cell line Bc as target cell.

† % reductions of target cell radioactivity are all significant by *t* test at $P < 0·001$.

TABLE IV.—*Induction of transplantation immunity by Sac virus**

| Pretreatment | Challenge with Sac( + ): Cell dose | Days after infection | No. of tumours/ No. of mice | Latent period in days |
|---|---|---|---|---|
| Sac virus | $5 \times 10^5$ | 12 | 2/6 | 6 |
| | $5 \times 10^4$ | | 0/6 | |
| None | $5 \times 10^5$ | | 6/6 | 6 |
| | $5 \times 10^4$ | | 6/6 | 6 |

* Derived from Sac cells after rescue with MLV.

producer cells is summarized in Tables II, III and IV. In a previous paper (Weiland *et al.*, 1978), the immunogenic properties of Sac( − ) and Sac( + ) cells were reported in detail. It was shown that Sac( − ) cells gain the capacity to induce an immune response only after rescue leading to virus production. Therefore, only Sac virus and Sac producer cells are compared in parallel. Table II shows the presence of C′-dependent cytotoxic antibodies after virus injection as well as after producer-cell transplantation. The tumour stage of cell-induced tumours seemed to influence the antibody titres. Cytotoxic spleen cells were induced in tumour-bearers 12 days after virus injection or 12 days after transplantation of different cell doses of producer cells (Table III). Transplantation protection against challenge with Sac( + ) cells could be induced by Sac virus (Table IV) as well as by Sac(+) cells, as reported in a previous communication (Weiland *et al.*, 1978).

*Events occurring after infection of MEF with sarcoma virus or co-cultivation of MEF with transformed Sac virus-producing cells.*—After infection with sarcoma virus (Sac virus or MSV-B-62) the morphology of the whole MEF-culture was altered, starting about 60 h p.i. This state, in which cells showed a morphology characteristic of transformed cells, lasted only 8–10 days. After the second weekly transfer the cultures already resembled the mock-infected controls. Rapp & Todaro (1978) call this event transient transformation.

In co-cultures of MEF with Sac(+) cells, foci of round to fusiform cells were observed developing on the MEF monolayer in the first week. Thereafter, cultures grew out resembling more and more the

FIG. 2.—Co-culture of mouse embryo fibroblasts (MEF) with stably transformed Sac(+) cells.

(a) A few densely stained round cells and spindle cells surrounded by normal cells one week after start of the co-culture (ratio MEF to Sac(+) cells: 20,000:1).
(b) One week after the first passage the portion of densely stained cells increases.
(c) One week after the second passage the densely stained cells predominate almost entirely over normal cells.
Haematoxylin and eosin. ×85.

original transformed cell culture. Fig. 2 shows 3 stages of the co-culture. Densely stained round cells and spindle cells were at first only sparsely seen, but finally preponderated over cells with the normal phenotype.

Remarkable differences were seen when the results of soft-agar assays, virus assays, and tumour induction were compared. In contrast to the co-culture that regularly induced colonies in soft agar, sarcoma virus-infected MEF were unable to form colonies. The co-culture produced sarcoma virus in addition to helper virus over the whole observation period of 12 transfer generations. The sarcoma-virus-infected MEF, however, released sarcoma virus and helper virus only for 2–3 weeks; during the following 9 transfer generations only helper virus was demonstrable. Tumour induction by transplantation of sarcoma-virus-infected MEF could be observed 1–2 weeks after infection, leading to tumours which always regressed. Tumour induction by transplantation of co-cultured MEF and Sac(+) cells led to tumours which showed both ultimately progressive and regressive growth, a behaviour characteristic of the original transformed culture.

### DISCUSSION

Although the MSV-M system has been studied for more than a decade the biology of this tumour is not yet fully understood. It is not certain whether MSV-M injection leads to tumour cells that are really autonomous, *i.e.* whether tumour development depends on the production of virus and continuous recruitment of newly infected transformed cell, or on the proliferation of transformed cells (Levy & Leclerc, 1977). In the avian system, tumours are believed to grow by recruitment of newly transformed cells by Rous-sarcoma-virus infection (Pontén, 1964).

Studies with mouse tumours induced by transplantation of stably transformed sarcoma-virus-producing cells on the one hand, and by injection of sarcoma virus alone on the other, may help to answer this question.

Recently, we reported on a non-producer tumour cell (Sac(−)) with rescuable sarcoma-virus genome derived from a recurring MSV-M-induced tumour (Weiland *et al.*, 1978). These Sac(−) cells are of low antigenicity and always induce progressively growing tumours in mice. In this respect they resemble the non-producer cells of Aaronson & Rowe (1970). After rescue with Moloney helper virus, Sac( – ) cells gained strong antigenicity (Sac( + ) cells) and induced a strong immune response in their hosts. In spite of this immune response, tumours with a progressive course are able to develop. Tumours resulting from injection of rescued sarcoma virus (Sac-virus tumours), however, gave rise to a strong immune response and always regressed. We were interested to know why the antigenic Sac( + )-cell tumour was able to grow progressively in spite of the existence of a strong immune response and why this never happened with Sac-virus tumours.

The following observations must be taken into account for the interpretation of the present results. The growth course of Sac( + )-cell tumours is obviously dependent on the number of cells used for injection in respect of the distribution of these cells in the tissue. If the inoculated cells find an appropriate microenvironment and are able to build up a dense agglomeration of tumour cells, a progressively growing tumour may arise that resists the immune defence mechanisms which develop within a few days. This resistance may be explained by mechanical prevention of immune factors from reaching most of those densely packed tumour cells. In this case tumour cells prevail in the tissue and tumours develop which are morphologically similar to Sac( – ) tumours. They are composed of compactly growing tumour cells accompanied by very little connective tissue. Division of the implanted tumour cells obviously predominates over new recruitment of transformed cells by viral infection.

Existence of immunosensitive tumour cells in progressively growing Sac( + )-cell tumours was recently reported (Weiland *et al.*, 1978). A tumour-cell suspension prepared from a progressively growing tumour was transplanted into normal and immune mice (pretreated with Sac virus). Whereas the control mice developed tumours, all immune mice remained free of tumours, independently of the transplanted cell concentration.

Regression of tumours after transplantation of low concentrations of Sac( + ) cells obviously occurs if virus-induced proliferation of fibroblastic tissue overwhelms growth of the typical tumour cells. This produces tumours in which the typical tumour cells are rarely visible, and which resemble in morphology and tumour course Sac-virus tumours. Immune destruction of tumour cells may be more successful in these tumours because they are more loosely arranged in the tissue.

Besides the active immune response seen in animals with Sac( + )-cell tumours and in animals with Sac-virus-induced tumours (Tables II, III, IV, and a recent report by Weiland *et al.* (1978)), a peculiarity of the transforming capacity of MSV-M, the transient transformation (Rapp & Todaro, 1978) of virus infected cells, seems to be important for the regular regression of sarcoma virus-induced tumours. In virus-induced tumours there is no histologically characteristic type of tumour cell. Holden *et al.* (1976) failed to demonstrate tumour cells in suspensions of MSV-M-induced tumours. We were not successful in isolating stably transformed cells from TEC or in inducing stably transformed cells by sarcoma-virus infection of MEF *in vitro*. In Sac( + ) as well as in Sac( – ) cell tumours, however, clonal proliferation occurs. From these tumours the cell of origin can easily be reisolated. The cultured cells arising from Sac( + )- and Sac( – )-cell-tumour explants resemble in their phenotype, growth in soft agar and their virological and tumour properties the cell strain used for the tumour induction.

Interest in the regression of tumours induced by MSV-M has almost exclusively focused on immunological events (Levy & Leclerc, 1977), whereas the role of the transforming capacity of MSV-M for the tumour growth has been neglected. To our knowledge, the only exception is a report by Penelli *et al.* (1975), in which the peculiar behaviour of MSV-M-infected cells is discussed as a reason for the regular regression of MSV-M-induced tumours, besides the existence of an immune response in tumour-bearing hosts. In this respect, it is interesting to note that Simons & McCully (1970) were not successful in establishing transplantable tumours after MSV-M infection, and that Simons (1970) did not succeed in inducing stably MSV-M-transformed mouse fibroblasts *in vitro*. According to Gazdar *et al.* (1976), most acutely MSV-M-infected mouse cells fail to divide, although many of them retain the ability to exclude Trypan blue.

Parkman *et al.* (1970) supposed that overgrowth of MSV-M-transformed MEF by helper-virus-infected MEF was the reason for the failure to establish MSV-M-transformed mouse cells, since MLV-M is usually found in excess in MSV stocks (Hartley & Rowe, 1966). However, the results of our co-culture studies with MEF and few Sac(+) cells do not support this assumption. The transformed Sac(+) cells overgrew MEF after 3 transfers at weekly intervals. Repetition of this experiment with MLV-M-infected MEF showed the same results (data not shown).

In special cases stable transformation of mouse cells by MSV-M can be achieved. By the use of particular cell strains such as BALB/3T3 and NIH/3T3 (Jainchill *et al.*, 1969), or by the use of particular virus strains such as the widely used S+L− strain (Bassin *et al.*, 1970; Aaronson *et al.*, 1972; Peebles *et al.*, 1975), that contains MSV-M in a greater amount than MLV-M. Though it is possible experimentally to obtain stably MSV-M-transformed mouse cells, it seems to be a rare event (Bassin *et al.*, 1970). The ratio of MSV-M-induced foci to soft-agar-colony-forming units was in excess of 1000 to 1, compared to a ratio of 50 to 1 obtained with the B-34 virus derived from MSV-Harvey-induced hamster tumour cells (Zavada & Macpherson, 1970).

The motive for our studies was the question: why do two tumours which induce the same immune response in their hosts differ in the course they run? The data reported here suggest that the fate of a tumour is not only dependent on its induced immune response but also on its growth properties. An MSV-M tumour regularly shows regression due to the strong immune response it induces and the fact that MSV-M infection only rarely, if at all, results in autonomous tumour cells. However, a tumour induced by transplantation of virus-producing (therefore strongly antigenic) autonomous cells is able to grow progressively in the presence of a strong immune response. This is assumed to be due to the mode of growth preventing intimate contact between the transplanted cells with immune defence mechanisms.

We are indebted to Dr J. H. Cox for reading the manuscript. We wish to thank Miss A. Schenk and Mr G. Balke for technical assistance.

REFERENCES

AARONSON, S. A. & ROWE, W. P. (1970) Nonproducer clones of murine sarcoma virus transformed BALB/3T3 cells. *Virology*, **42**, 9.

AARONSON, S. A., BASSIN, R. H. & WEAVER, C. (1972) Comparison of murine sarcoma viruses in nonproducer and +-transformed cells. *J. Virol.*, **9**, 701.

BASSIN, R. H., TUTTLE, N. & FISCHINGER, P. J. (1970) Isolation of murine sarcoma virus transformed mouse cells which are negative for leukemia virus from agar suspension cultures. *Int. J. Cancer*, **6**, 95.

BATHER, R., LEONARD, A. & YANG, J. (1968) Characteristics of the *in vitro* assay of murine sarcoma virus (Moloney) and virus-infected cells. *J. Natl Cancer Inst.*, **40**, 551.

BERMAN, L. D. & ALLISON, A. C. (1969) Studies on murine sarcoma virus; a morphological comparison of tumorigenesis by the Harvey and Moloney strains in mice, and the establishment of tumor cell lines. *Int. J. Cancer*, **40**, 820.

COLLAVO, D., COLOMBATTI, A., BIASI, G., CHIECO-BIANCHI, L. & DAVIES, A. J. S. (1976) Immune reactivity in the Moloney strain of murine sarcoma virus oncogenesis: Requirement of thymus-derived lymphocytes for *in vivo* protection. *J. Natl Cancer Inst.*, **56**, 603.

DAVIS, S. (1975) Moloney sarcoma virus-induced tumors in athymic (nude) mice: growth pattern and antibody responses. *J. Natl Cancer Inst.*, **54**, 793.

FEFER, A., MCCOY, J. L. & GLYNN, J. P. (1967) Induction and regression of primary Moloney sarcoma virus-induced tumors in mice. *Cancer Res.*, **27**, 1626.

GABBIANI, G., HIRSCHEL, B. J., RYAN, G. B., STATKOV, P. R. & MAJNO, G. (1972) Granulation tissue as a contractile organ. A study of structure and function. *J. Exp. Med.*, **135**, 719.

GAZDAR, A. F., STULL, H. B., KILTON, L. J. & BACHRACH, U. (1976) Increased ornithine decarboxylase activity in murine sarcoma virus infected cells. *Nature*, **262**, 696.

HARTLEY, J. W. & ROWE, W. P. (1966) Production of altered cell foci in tissue culture by defective Moloney sarcoma virus particles. *Proc. Natl Acad. Sci., U.S.A.*, **55**, 780.

HERBERMAN, R. B. (1977) Immunogenicity of tumor antigens. *Biochim. Biophys. Acta*, **473**, 93.

HOLDEN, H. T., HASKILL, J. S., KIRCHNER, H. & HERBERMAN, R. B. (1976) Two functionally distinct anti-tumor effector cells isolated from primary murine sarcoma virus-induced tumors. *J. Immunol.*, **117**, 440.

JAINCHILL, J. L., AARONSON, S. A. & TODARO, G. J. (1969) Murine sarcoma and leukemia viruses: assay using clonal lines of contact-inhibited mouse cells. *J. Virol.*, **4**, 549.

LAW, L. W., TING, R. C. & STANTON, M. F. (1968) Some biologic, immunogenic, and morphologic effects in mice after infection with a murine sarcoma virus. I. Biologic and immunogenic studies. *J. Natl Cancer Inst.*, **40**, 1101.

LEVY, J. P. & LECLERC, J. C. (1977) The murine sarcoma virus induced tumor: exception or general model in tumor immunology? *Adv. Cancer Res.*, **24**, 1.

PARKMAN, R., LEVY, J. A. & TING, R. C. (1970) Murine sarcoma virus: The question of defectiveness. *Science*, **168**, 387.

PEEBLES, P. T., GERWIN, B. I., PAPAGEORGE, A. G. & SMITH, S. G. (1975) Murine sarcoma virus defectiveness. Viral polymerase expression in murine and nonmurine host cells transformed by S+L− type murine sarcoma virus. *Virology*, **67**, 344.

PENELLI, N., CHIECO-BIANCHI, L., COLLAVO, D. & CECCHETTO, A. (1975) Studio istopatologico e ultrastrutturale dei tumori indotti da virus del sarcoma murino (MSV). *Tumori*, **61**, 129.

PONTÉN, J. (1964) The *in vivo* growth mechanism of avian Rous sarcoma. *Natl Cancer Inst. Monogr.*, **17**, 131.

RAPP, U. R. & TODARO, G. J. (1978) Generation of new mouse sarcoma viruses in cell culture. *Science*, **201**, 821.

ROWE, W. P., PUGH, W. E. & HARTLEY, J. W. (1970) Plaque assay techniques for murine leukaemia viruses. *Virology*, **42**, 1136.

SIEGLER, R. (1970) Pathogenesis of virus-induced murine sarcoma. I light microscopy. *J. Natl Cancer Inst.*, **45**, 135.

SIMONS, P. J. (1970) The behaviour of two strains of murine sarcoma virus *in vitro*. *Aust. J. Exp. Biol. Med. Sci.*, **48**, 105.

SIMONS, P. J. & MCCULLY, D. J. (1970) Pathologic and virologic studies of tumors induced in mice by two strains of murine sarcoma virus. *J. Natl Cancer Inst.*, **44**, 1289.

STANTON, M. F., LAW, L. W. & TING, R. C. (1968) Some biologic, immunogenic, and morphologic effects in mice after infection with a murine sarcoma virus. II. Morphologic studies. *J. Natl Cancer Inst.*, **40**, 1113.

STUTMAN, O. (1975) Delayed tumor appearance and absence of regression in nude mice infected with murine sarcoma virus. *Nature*, **253**, 142.

TING, R. C. & BADER, A. V. (1969) A quantitative study on transformation of hamster embryo cells *in vitro* by murine sarcoma viruses (Harvey and Moloney). *Virology*, **39**, 194.

TOYOSHIMA, K. & VOGT, P. K. (1969) Temperature sensitive mutants of an avian sarcoma virus. *Virology*, **39**, 930.

WEIL, R. (1978) Viral tumor antigens. A novel type of mammalian regulator protein. *Biochim. Biophys. Acta*, **516**, 301.

WEILAND, E. & WEILAND, F. (1974) Transplantierbarer Aszitestumor aus einer Maus mit einem Moloneysarkom: Tumor-, Antigen- and Viruseigenschaften. *Z. Immunitaetsforsch.*, **148**, 151.

WEILAND, E. & MUSSGAY, M. (1975) Tumor inhibiting capacity of spleen and lymph node cells from mice with murine sarcoma virus (MSV-M)-induced tumors. *Z. Immunitaetsforsch.*, **150**, 414.

WEILAND, E. & MUSSGAY, M. (1976) Detection of cytotoxic lymphoid spleen cells from STU-mice with Moloney sarcoma by a $^{3}$H-proline microcytotoxicity assay. *Med. Microbiol. Immunol.*, **162**, 81.

WEILAND, E., MUSSGAY, M. & WEILAND, F. (1978) Nonproducer malignant tumor cells with rescuable sarcoma virus genome isolated from a recurrent Moloney sarcoma. *J. Exp. Med.*, **148**, 408.

ZAVADA, J. & MACPHERSON, I. (1970) Transformation of hamster cell lines *in vitro* by a hamster sarcoma virus. *Nature*, **225**, 24.